# Trauma facilities in Denmark - a nationwide cross-sectional benchmark study of facilities and trauma care organisation

**DOI:** 10.1186/s13049-018-0486-1

**Published:** 2018-03-27

**Authors:** Jesper Weile, Klaus Nielsen, Stine C. Primdahl, Christian A. Frederiksen, Christian B. Laursen, Erik Sloth, Ole Mølgaard, Lars Knudsen, Hans Kirkegaard

**Affiliations:** 1 Emergency Department, Regional Hospital Herning, Herning, Denmark; 2 Research Center for Emergency Medicine, Aarhus University Hospital, Nørrebrogade 44, building 1B, 8000 Aarhus C, Denmark; 3 Department of Medicine, Section of Respiratory Medicine, University Hospital Hvidovre, Hvidovre, Denmark; 4 Department of Cardiology, Aarhus University Hospital, Aarhus, Denmark; 5 Department of Respiratory Medicine, Odense University Hospital, Odense, Denmark; 6 University of Cape Town, Cape Town, South Africa; 7 Emergency Department, Aarhus University Hospital, Aarhus, Denmark; 8 Department of Anesthesiology and Intensive Care, Aarhus University Hospital, Aarhus, Denmark

## Abstract

**Background:**

Trauma is a leading cause of death among adults aged < 44 years, and optimal care is a challenge. Evidence supports the centralization of trauma facilities and the use multidisciplinary trauma teams. Because knowledge is sparse on the existing distribution of trauma facilities and the organisation of trauma care in Denmark, the aim of this study was to identify all Danish facilities that care for traumatized patients and to investigate the diversity in organization of trauma management.

**Methods:**

We conducted a systematic observational cross-sectional study. First, all hospitals in Denmark were identified via online services and clarifying phone calls to each facility. Second, all trauma care manuals on all facilities that receive traumatized patients were gathered. Third, anesthesiologists and orthopedic surgeons on call at all trauma facilities were contacted via telephone for structured interviews.

**Results:**

A total of 22 facilities in Denmark were found to receive traumatized patients. All facilities used a trauma care manual and all had a multidisciplinary trauma team. The study found three different trauma team activation criteria and nine different compositions of teams who participate in trauma care. Training was heterogeneous and, beyond the major trauma centers, databases were only maintained in a few facilities.

**Conclusion:**

The study established an inventory of the existing Danish facilities that receive traumatized patients. The trauma team activation criteria and the trauma teams were heterogeneous in both size and composition. A national database for traumatized patients, research on nationwide trauma team activation criteria, and team composition guidelines are all called for.

**Electronic supplementary material:**

The online version of this article (10.1186/s13049-018-0486-1) contains supplementary material, which is available to authorized users.

## Background

Trauma is the leading cause of death in the Western world among adults aged < 44 years [1]. The incidence of major trauma ranges from 30 to 52 per 100,000 inhabitants per year in all of Scandinavia [2]. During 2015, public hospitals in Denmark treated a total of 416,309 patients for injuries; a total of 84,762 patients were hospitalized due to acute emergencies and grievous bodily harm [3]. The number of these patients who were received by trauma teams remains unknown.

The civil trauma system in Europe in general and Denmark in particular was adapted from the American system introduced in the 1970s [4, 5]. Evidence supports the centralization of trauma care and the use of multidisciplinary trauma teams during the initial assessment and treatment of traumatized patients [6–8].

The Danish healthcare system is in a state of constant development. Highly specialized major trauma centers are distributed throughout the nation’s four largest cities although a number of minor facilities also handle traumatized patients. Trauma care is handled solely by public hospitals in Denmark; in 2004, a total of 55 hospitals cared for traumatized patients, and trauma team training was in the early stages of development [9]. Data from cross-sectional surveys of trauma care exist from other countries [10, 11] although descriptions in the literature of the total number and organization of trauma facilities in Denmark remain lacking.

While different recommendations on the composition of trauma teams have been put forth [1, 12], no evidence exists that any one specific trauma team composition is superior to others. Internationally, the composition varies significantly from one country to another and even within countries [1]. Consensus is also lacking on which criteria should be used to activate a trauma team although several different criteria have been proposed [2, 13, 14]. While studies in the literature on the national organization of in-hospital cardiac arrest teams in Denmark exist [15], to the best of our knowledge, no descriptions have been put forth in the literature of the compositions of trauma teams in Denmark or of the activation criteria they use.

More detailed data on current standards is called for in order to optimize and unify the national and international organization of trauma patient management. Knowledge of the current state is paramount to facilitate development in the field. This knowledge will benefit not only the national community, but also inspire internationally in comparable countries. Thus, this study aimed to identify all trauma facilities in Denmark and to present the geographical distribution across the country. Its secondary aim was to investigate any differences in the organization of management of traumatized patients during all in hospital steps, from the trauma team activation criteria to the composition of the trauma team as well as training of trauma teams, the establishment of audits and databases of trauma care.

## Methods

The study was designed as a cross-sectional observational study performed in three phases, as follows.

### Identification of trauma facilities

The identification of trauma facilities was performed by using an online database [16] administered by the Danish Regions, Local Government Denmark (Danish: Kommunernes Landsforening (KL)), and the Danish Ministry of Health; this database displays a complete list of all public hospitals and treatment facilities in Denmark. The Danish Ministry of Health oversees the validity of the list. No private hospitals in Denmark handle traumatized patients. Once the complete list of all public hospitals was acquired we contacted each hospital by telephone and inquired whether the hospital received acutely injured patients by ambulance (road, air or ship) at the time of contact and, if so, whether the hospital had a formalized trauma team.

### Gathering guidelines

We gathered all local guidelines for the assessment of trauma patients by doing an online search of all publicly available guidelines. The five Danish Regions each have one specific website containing all guidelines for public hospitals. (The public websites that were used are listed in Additional file 1). Searches were conducted for the phrases “trauma manual” and “trauma” in the selected platforms. If guidelines were unavailable or were older than 2015, emails were sent to the emergency department and the orthopedic department to ensure that the most recent guidelines had been obtained. We conducted the search in 2016 and chose 2015 as cutoff allowing an annual update to be considered recent.

### Telephone interviews

To strengthen the study we conducted phone interviews with personnel involved in the trauma team. We called all hospitals included in phase one, with a request to speak to the person who generally conducts referrals of traumatized patients as this person was expected to have detailed knowledge of the local organisation. In most facilities, this would be a nurse via the hospital referral. This person would then be interviewed in a structured manner on the organization of the trauma team and the trauma activation criteria. (For the interview guide, see Additional file 1)..

Phone calls were also made to an attending orthopedic surgeon and an attending anesthesiologist on call from each facility, as we expected these specialists to be members of the trauma teams in all facilities. These people were interviewed in a structured manner on the organizational aspects of the trauma team. (All interview guides may be found in Additional file 1).

All phone calls were conducted during weekdays from 9:00 a.m. to 8 p.m. If the team members or personnel were unavailable, the phone call was repeated seven times at different times of the day. After the seventh missed call, an email containing the structured interview was sent to the department. This email was followed up by a reminder email. If no response was forthcoming, then the department was categorized as not responding.

Telephone interviews where supervised by HK, professor in Emergency Medicine. All interviews where conducted by JW, SCP, KN or Stig Holm Jensen (BS in medicine). Interview guides were drafted by JW. The interviews where practiced where SCP called JW and conducted a “pilot interview” to omit ambiguous wording. The final version of the interviews were approved by all authors and conducted in a structured manner according to the Interview Guide (see Additional file 1).

In the case of any disagreements about the participants in a trauma team, we used the description from the most relevant specialist; for example, an anesthesiologist’s assertion would be weighted over others’ opinions regarding the number of anesthesiologists or anesthesia nurses who would be present at a trauma team activation.

### Other resources

In order to evaluate any correlation between numbers of members in the trauma team and hospital size, we used the number of beds in the hospital as a surrogate marker for hospital size. Hospitals were classified as large (> 600 beds), major (400–600 beds), minor (200–400 beds), or small (< 200 beds) (Definition from Lauridsen et al. [15]). These numbers are based on data obtained from the Danish Health Authority on 01.04.2016. (All hospital sizes may be viewed in Additional file 1).

## Definitions

The following definitions were created and followed for the study.

- Trauma center: a highly specialized unit as defined by the Danish Health Authority. Denmark has four trauma centers. Patients expected to have major trauma are referred to these centers by the pre hospital service thereby bypassing local facilitates. The description of pre hospital triage is beyond the scope of this paper.
- Trauma Team Activation: the initiation of a certain procedure in which prespecified Trauma Team consisting of personnel from multiple specialties with predefined specific tasks are summoned to the trauma room to initiate the care of the acutely injured patient.
- Trauma facility: a hospital, no matter the size, that receives traumatized patients. The four trauma centers in Denmark are also considered trauma facilities, but highly specialized.
- Audit: A formalized prescheduled review of all Trauma Team Activation. This does not include ad hoc debriefing in severe cases.
- Trauma Patient: A patient where the in hospital Trauma Team is activated.

## Statistical analysis

All variables and the compositions of teams are presented in actual numbers and percentages; medians and interquartile ranges (IQRs) are presented as non-parametric data. The Kruskal-Wallis test was applied to compare groups of hospitals and number of team members. Intersubject reliabilities are presented as percentages. All data analyses were performed using STATA 13 (StataCorp, College Station, TX, USA).

## Results

**Fig. 1:**
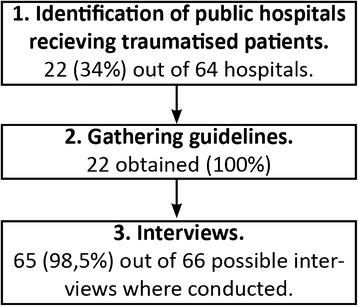
Overview of the study setting

Data was collected between August and December 2016. Figure 1 shows the three phases of the study. We identified 64 public hospitals in Denmark whereof 22 hospitals received traumatized patients; All hospitals (22/22 = 100%) that received traumatized patients had a multidisciplinary trauma team. All 22 (100%) hospitals had a trauma care manual; we retrieved 10 from publicly available Internet websites and the remaining 12 were retrieved via e-mail. The trauma care manuals were of different ages as shown in Table 1.

**Table 1:** Trauma Care Manuals retrieved and year of update

Year	*n* (%)
2009	1 (4,5)
2010	1 (4,5)
2011	1 (4,5)
2012	3 (13,6)
2013	2 (9,1)
2014	–
2015	5 (22,7)
2016	9 (40,9)

### Geographical distribution

**Fig. 2:**
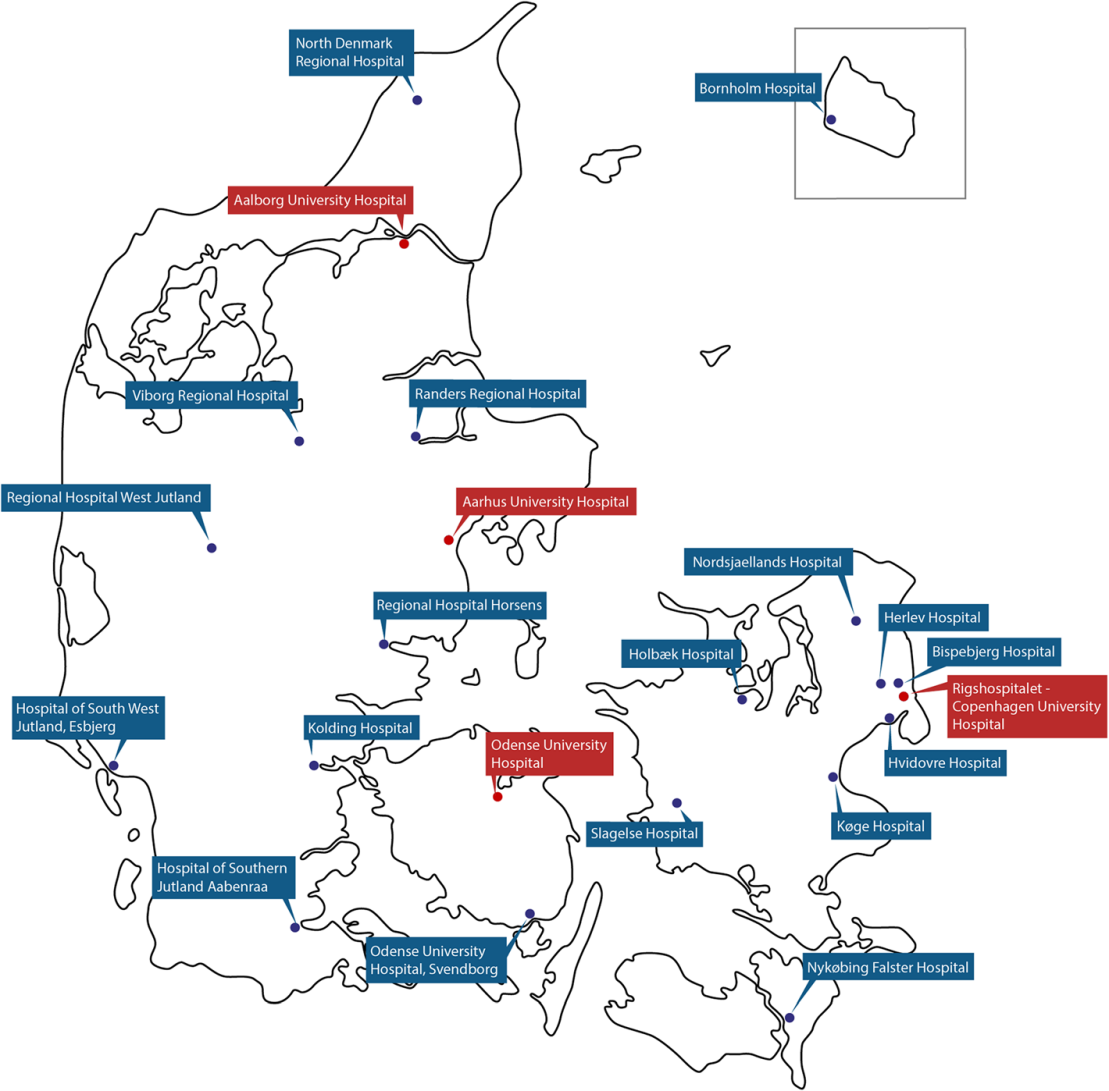
Map showing the distribution of the different trauma facilities in Denmark. The trauma centers are marked with a red dot

The geographical distribution of the hospitals is shown in Fig. 2. All 22 trauma facilities were contacted by telephone. Out of 66 planned telephone interviews, 65 (98.5%) were completed. One (1.5%) orthopedic department declined to participate.

### Inter subject reliability

Inter subject reliability was calculated on all questions regarding personnel present between the three subjects interviewed: The anesthesiologist, the orthopedic surgeon and the referral nurse. The overall reliability was 56,3%. Inter subject agreement on physicians present was 62,5% and inter rater agreement on which specialty was trauma leader was 95,5%. Overall the reply “do not know” was given in 10,7% of answers. Out of all “do not know” answers where given in 93,5% of all cases to questions regarding personnel other than own specialty (e.g. The orthopedic surgeon asked about the number of anesthesiology nurses present). Agreement between referral nurse and orthopedic doctor on orthopedic personnel was 90,9%. And agreement between the referral nurse and the anesthesiologist on anesthesiology personnel included in the trauma team was 95,6%.

### Trauma team activation criteria

All facilities had formalized trauma team activation criteria although the criteria were heterogeneous throughout the country. The guidelines may be divided into two main types. In the first type, a scoring system is used where points are accredited to certain types of injury; the trauma team is called if a certain number of points is reached (see Table 2).

**Table 2:** An example of the most widely distributed trauma activation criteria used in Denmark

Trauma Team Activation Criteria type 1				
Trauma Team Activation is triggered if the patient scores 2 points or more				
	0	1	2	Points
Trauma mechanism	Low-energy	High-energy^a^		
Respiration	Normal	Labored	Respiration stop	
Circulation	BP > 90 mmHg		BP < 90 mmHg	
Level of consciousness	Awake	Confused	Unconscious	
Thorax	Indolent	Pain	Open lesion	
Abdomen	Indolent	Pain	Open lesion	
Cervical region and back	Indolent	Pain	Open lesion	
Arms, legs, and pelvis	Indolent	Open lesion		
Sum				

^a^Definition of high-energy trauma: Fall > 3 x own height - Pedestrian hit by car - Death in same vehicle - Ejection from vehicle - Stuck in vehicle - Deformity of vehicle - Vehicle rolled over - Penetrating lesion

In the second type, the system is divided into three categories: the anatomical criteria, physiological criteria, and mechanism of trauma. In this activation protocol, a series of variables under each criterion could individually trigger the trauma team activation if the patient has suffered a relevant trauma (see Table 3).

**Table 3:** An example of the second most widely distributed trauma activation criteria used in Denmark

Trauma Team Activation Criteria type 2
Trauma Team Activation is triggered if the answer is yes to any of the following
Anatomical criteria
Penetrating lesions
Flail chest
Fracture to more than two long tubular bones or suspected pelvic fracture
Suspicion of fracture to the spine
Amputation proximal to wrist or ankle
Suspected internal hemorrhage
Burns (children > 10%, adults > 15%)
Physiological criteria
Change in respiration (dyspnea, tachypnea, bradypnea)
Hypotension < 90 mmHg
Change in mental status (GCS < 13)
Mechanism
Traffic accident with deformity of vehicle
Patient ejected from vehicle
Death in same vehicle
Motorcycle / bike / moped crash > 30 km/h
Auto vs. pedestrian
Fall from more than 4 m
Child fall from more than 3 x own height
Drowning or hypothermia (< 32 degrees C)

Out of 22 trauma facilities, 15 (68.2%) used a point scoring system, while 6 (22.7%) used a system similar to that exemplified in Table 3. The remaining facility (4.5%) used a mixed system that listed targeted variables (e.g., systolic blood pressure < 90 mmHg) that were strong enough to activate trauma directly; other types of injury also provided points that would also activate the trauma team activation. One trauma center reported using a two-tiered system where two different trauma teams (consisting of a basic team and an extended team) were used. This meant that, according to the expected severity of the injuries, either a basic or an extended trauma team could be summoned. We used the basic trauma team composition for comparison with other teams.

### Trauma leader

**Fig. 3:**
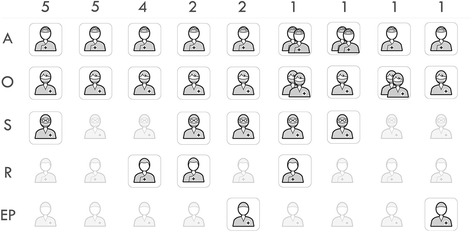
The figure shows the different trauma teams’ compositions and the number of hospitals with any given composition; only the physicians are included in the Fig. A: anesthesiologist; O: orthopedic surgeon; S: abdominal surgeon; R: radiologist; EP: emergency physician

In 14 (63.6%) of the facilities, the participating orthopedic surgeon was the trauma leader, while in 8 (36.4%) facilities, the trauma leader was the anesthesiologist. An abdominal surgeon was included in the trauma team in 10 (45.5%) of the hospitals; the remaining 12 (54.5%) did not have such surgeons. All facilities that received traumatized patients had access to surgical facilities and an abdominal surgeon on call. One (4.5%) facility did not receive traumatized patients during the night because the orthopedic surgeon was only present at the hospital during the day, from 7 a.m. to 7 p.m. Figure 3 shows the different compositions of the trauma teams and which physicians participated.

### Total personnel on trauma team

In addition to the physicians involved in the trauma team, other participants were present as well. The median size of the trauma team, including all staff, was 10 (IQR 10–12). The smallest team consisted of 9 people, and the largest was 17. Table 4 shows the different roles of participants and number of facilities in which they are involved in the management of traumatized patients. The Kruskal-Wallis test was performed to assess the relationship between the size of the trauma facility and the number of participants on the trauma team. No correlation was found between number of participants on the trauma team and the number of beds (*p* = 0.15).

**Table 4:** Total number of staff in trauma teams in the 22 facilities in Denmark. The columns represent the number of a certain kind of staff member on the trauma team. For example, 17 facilities have one anesthesiology nurse in the trauma team, while five facilities have two

Non-physician staff included in trauma teams; n (%)	0	1	2	3
Anesthesiology nurse	–	17 (77.3)	5 (22.7)	–
Nurse from the emergency department	–	1 (4.5)	20 (90.9)	1 (4.5)
Medical laboratory technician	1 (4.5)	19 (86.4)	2 (9.1)	–
Orderly	–	10 (45.5)	12 (54.5)	–
Secretary	4 (18.2)	18 (81.8)	–	–
Radiographer / nurse from the radiology department	–	18 (81.8)	4 (18.2)	–

### Database and simulation

A database of trauma activations was maintained in 9 (40.9%) of the facilities. Two (9.1%) facilities performed regular audits on their trauma management; 20 (90.9%) conducted simulation training, 35.0% of which used video for simulation training. An overview of the databases, audits, and simulations is shown in Table 5.

**Table 5:** Questions about databases, audits, and simulations

Databases, audits, and simulations	Yes	No	Unknown
Do you keep a database on traumas?	9 (40.9)	8 (36.4)	5 (22.7)
Do you perform regular audits on traumas?	2 (9.1)	20 (90.9)	–
Do you conduct regular simulation training?	20 (90.9)	2 (9.1)	–
Do you use video for simulations?^a^	7 (35.0)	6 (30.0)	7 (35.0)

^a^Only applicable to facilities that perform simulation training

Simulation training was performed in 20 (90.9%) hospitals; out of 14 facilities in which it was possible to establish a figure, the median annual number of training sessions was 2 (IQR 2–3).

## Discussion

This nationwide cross-sectional benchmark study presents the 22 trauma facilities that cared for traumatized patients in Denmark in 2016. Our study revealed at least three different trauma activation criteria and nine different compositions of trauma teams, with team sizes ranging from 9 to 17 participants. Databases were maintained in nine (40.9%) facilities, while two (9.1%) facilities performed regular audits of trauma. Simulation training was performed in 20 (90.9%) of the total facilities.

The twenty-two facilities we located represent half of the 55 facilities reported in 2004 [9]; this number is in concordance with the centralization of hospitals during the last decade as well as developments during that time in Norway [10]. A previous study has established that the incidence of major trauma in Scandinavian countries ranges from 30 to 52 per 100,000 inhabitants per year [2]. Out of 5.7 million inhabitants in Denmark, this figure would result in approximately 1700–3000 major traumas per year. These traumas will not be evenly distributed among the facilities, as the four major trauma centers are expected to handle the majority of these cases. While it is beyond the scope of this paper to discuss the appropriate number of trauma centers for the country in total, our finding of 22 facilities shows that minor hospitals will be expected only to handle a small number of major traumas per year.

We found no correlation between number of physicians on the trauma team and the size of the trauma facility. Kelleher et al. have suggested that trauma team size follows “the principle of diminishing marginal returns.” This means that the efficiency of a trauma team will only increase until a certain threshold; after that point, its efficiency will decrease if more people are added to the team [17]. The current study was performed in a pediatric setting and found that the optimal size was a total of 13 team members. Because the teams in the current study vary from 9 to 17 members, future research is required to determine the most effective size of a trauma team for adult populations.

One facility in our study reported having a two-tiered trauma team. Earlier works have demonstrated that having two levels of trauma teams can be efficient [14]. The facility that uses a two-tiered system in our study has a basic as well as an extended trauma team. Two-tiered systems have been reported abroad, where the first tier consists of an emergency physician or an orthopedic surgeon and nurses from the emergency department. This arrangement facilitates in-hospital assessment and the team can always assemble the larger team if necessary. This structure improves triage capabilities and minimizes cost; in addition, studies have demonstrated that the use of a two-tiered trauma team activation criteria system can discriminate the severity of trauma [18, 19]. A previous Danish study has reported profound over-triage when using a one-tiered system [20]; on this basis, we encourage the implementation of clinical trials that would focus on the application of a small low-level trauma team within a multi-tiered system.

Because two trauma team activation criteria exist, it is reasonable to believe that one performs more precisely than the other. The American College of Surgeons recommends using a system based on physiologic criteria, anatomic criteria, and mechanism of injury [21]; the system that is most widely used in Denmark is very close to this recommendation. We recommend that a national consensus on evidence-based uniform criteria should be reached.

Only a few hospitals use trauma databases, which makes it impossible to estimate the total number of trauma team activations retrospectively. Moreover, this makes it impossible to gather information on who or what triggered the trauma team activation for each patient. Although earlier papers have called for a national database for traumatized patients [22], such a database has yet to be established in Denmark. A national database would enable the precise quantification of over- and under-triage and would facilitate the use of evidence-based guidelines in the future. An assessment of two different activation criteria would then become possible according to over- and under-triage based on a standardized score such as the Injury Severity Score.

The use of simulation-based training in local facilities has demonstrated improvements to the management of trauma in many domains [23]. The auditing of a trauma team’s performance through the video review of simulations has shown the reductions of overall assessment time, time to intervention, and increased compliance with Advanced Trauma Life Support (ATLS) guidelines [24]. Hence scheduled simulation-based training and scheduled auditing of trauma team performance should be considered when revising guidelines; we encourage all facilities to introduce regular auditing and simulation training. Since live video recordings have been shown to be beneficial in reviewing actual traumas, this aspect should also be considered [24].

### Limitations

This study does have certain limitations. The results from the telephone interviews may have differed if another day had been chosen and a different physician had answered the call, both of which circumstances would affect the data in connection to the degree of training of the participants in the trauma team. We report a low overall intersubject reliability. However there is a very high intersubject reliability when looking at the subject’s knowledge of own specialty personell present. This is why we report the data from the most relevant person’s assertion. The answers could have been influenced by recollection bias, as the interviewees may have recalled an incorrect number e.g. number of simulations conducted per year. This study is also limited to a national survey that only covers Denmark. Despite these limitations, our study method can provide a framework for analysis of the trauma organization in other countries or institutions.

## Conclusion

In Denmark, trauma team activation criteria - as well as the trauma teams themselves - are heterogeneous in both size and composition. National consensus on trauma team activation criteria and team composition should be pursued. Future prospective studies should evaluate the superiority (and inferiority) of specific trauma team compositions, triage, organization, and training regimes.

## Additional file

Additional file 1: Appendix. (DOCX 26 kb)

## Abbreviations

ATLS: Advanced Trauma Life Support
IQR: Interquartile ranges
KL: Local Government Denmark (Danish: Kommunernes Landsforening)
