# Supplementary material for: Trauma facilities in Denmark - a nationwide cross-sectional benchmark study of facilities and trauma care organisation
Source: Scand J Trauma Resusc Emerg Med. 2018 Mar 27;26:22. doi: 10.1186/s13049-018-0486-1 (PMC5870211; doi:10.1186/s13049-018-0486-1)
Supplement: Supplementary file 1 — Appendix. (DOCX 26 kb) [file 13049_2018_486_MOESM1_ESM.docx]

**Additional file 1**

1. Websites containing publicly available guidelines for different regions in Denmark.

| **Websites containing guidelines for public hospitals in Denmark** |  |
| --- | --- |
| **The North Denmark Region:** | https://pri.rn.dk |
| **Central Denmark Region:** | http://e-dok.rm.dk |
| **Region of Southern Denmark:** | http://ekstern.infonet.regionsyddanmark.dk |
| **Region Zealand:** | http://dok.regionsjaelland.dk |
| **Capital Region:** | http://vip.regionh.dk |

**2. Interview guide**

Questionnaire

Date: ____ / ____ / _____, Time: _____ : _____ / Investigator: ______________________________

Hospital: _____________________________ / Department: ________________________________

Person interviewed:

🞐: Trauma leader 🞐: Other: __________________

🞐: Gives oral informed consent to participate in the study

Name: _______________________________ e-mail: ______________________@_____________________

🞐: House Officer 🞐: Senior House Officer 🞐: Specialist 🞐: Consultant

Trauma experience (participation in number of traumas during the last year):

🞐: 0 🞐: 1-10, 🞐: 11 – 30 🞐: 31-100 🞐: 101-300 🞐: >300 🞐: Do not know

Course you have participated in or certified in:

🞐: ALS 🞐: ATLS 🞐: PHTLS 🞐: Other: ____________________

Trauma activation:

Are you aware which criteria are used in your facility to activate the trauma call?:

🞐: Yes 🞐: No 🞐: We do not have criteria (If yes: Criteria must be forwarded by e-mail)

**In the trauma room**

Are there multiple types of trauma call? 🞐: Yes 🞐: No 🞐: Do not know

**Trauma call (Who is present. More than one x if more than one person)**

(🞐HO=House officer, 🞐SHO=Senior House Officer, 🞐Specialist)

Person: Educational level: Who is trauma leader

Orthopaedic Surgeon: 🞐HO, 🞐SHO, 🞐Specialist 🞐

Emergency physician 🞐HO, 🞐SHO, 🞐Specialist 🞐

Anaesthesiologist 🞐HO, 🞐SHO, 🞐Specialist 🞐

General surgeon 🞐HO, 🞐SHO, 🞐Specialist 🞐

🞐: Anaestesiology nurse (No: _____)

🞐: Nurse from emergency department (No: _____)

🞐: Medical Laboratory Technician 🞐: Orderly (No: ____) 🞐: Secretary

🞐: Radiologist 🞐: Radiology doctor

Other specialties present: (🞐HO=House officer, 🞐SHO=Senior House Officer, 🞐Specialist)

🞐: Paediatrician 🞐: Neuro surgeon 🞐: Thoracic surgeon

🞐HO, 🞐SHO, 🞐Specialist 🞐HO, 🞐SHO, 🞐Specialist 🞐HO, 🞐SHO, 🞐Specialist

🞐: Other Specialties: ____________________________________________________🞐HO, 🞐SHO, 🞐Specialist

**Registration of traumas:**

Do you keep a database on traumas?

🞐: Yes 🞐: No 🞐: Do not know

Do you perform regular audits on traumas?

🞐: Yes (If yes: How often: _________) 🞐: No 🞐: Do not know

🞐: Only on special occations: ___________________________

Do you conduct regular simulation training?

🞐: Yes (If yes: How often: _________) 🞐: No 🞐: Do not know

Do you use video for simulation?

🞐: Yes 🞐: No 🞐: Do not know

3. Hopitals listed with key figures of trauma team size and number of beds as a surrogate marker for size of hospital.

| **Hospital #** | **Physicians** | **Non Physicians** | **All** | **Trauma team Leader** | **No. of beds** |
| --- | --- | --- | --- | --- | --- |
|  |  |  |  |  |  |
| 1 | 4 | 7 | 11 | 1 | 252 |
| 2 | 3 | 7 | 10 | 1 | 688 |
|  |  |  |  |  |  |
| 3 | 3 | 6 | 9 | 1 | 393 |
| 4 | 2 | 9 | 11 | 1 | 232 |
| 5 | 3 | 7 | 10 | 1 | 236 |
| 6 | 3 | 7 | 10 | 1 | 528 |
| 7 | 3 | 9 | 12 | 1 | 902 |
|  |  |  |  |  |  |
| 8 | 2 | 8 | 10 | 1 | 353 |
| 9 | 3 | 8 | 11 | 1 | 972 |
| 10 | 2 | 8 | 10 | 1 | 127 |
| 11 | 3 | 9 | 12 | 1 | 301 |
| 12 | 2 | 8 | 10 | 1 | 355 |
|  |  |  |  |  |  |
| 13 | 3 | 7 | 10 | 2 | 317 |
| 14 | 4 | 6 | 10 | 1 | 282 |
| 15 | 2 | 8 | 10 | 1 | 268 |
| 16 | 3 | 7 | 10 | 2 | 322 |
|  |  |  |  |  |  |
| 17 | 4 | 9 | 13 | 2 | 98 |
| 18 | 3 | 8 | 11 | 2 | 580 |
| 19 | 4 | 8 | 12 | 2 | 579 |
| 20 | 6 | 11 | 17 | 2 | 1242 |
| 21 | 4 | 8 | 12 | 2 | 784 |
| 22 | 3 | 7 | 10 | 2 | 493 |

*Number of physicians, non-physicians, total and trauma team leader. 1=orthopedic surgeon, 2=anaestesiologist. Number of beds is in the hospital are based on own calculations made on numbers from the Danish Health Authority on 01.04.2016.*
